# A Comparative analysis of PESC and PPSC copolyesters: Insights into viscosity, thermal behavior, crystallinity, and biodegradability

**DOI:** 10.1016/j.heliyon.2024.e24728

**Published:** 2024-01-13

**Authors:** A. Kesavan, T. Rajakumar, M. Karunanidhi, A. Ravi, P.A. Vivekanand, P. Kamaraj, Natarajan Arumugam, S. Hari Kumar, Karthikeyan Perumal, Sinouvassane Djearamane, Mohammod Aminuzzaman, Ling Shing Wong, Saminathan Kayarohanam

**Affiliations:** a Department of Chemistry, Kalaignar Karunanidhi Government Arts College, Thiruvannamalai, India; b Department of Chemistry, Government Arts College, Udumalpet, India; c Centre for Catalysis Research, Department of Chemistry, Saveetha Engineering College, Thandalam, Chennai-602105, India; d Department of Chemistry, Bharath Institute of Higher Education and Research (BIHER), Chennai 600073, India; e Department of Chemistry, College of Science, King Saud University, P.O. 2455, Riyadh 11451, Saudi Arabia; f Chemistry Division, Department of Humanities and Science, Rajalakshmi Institute of Technology, Chennai 600124, Tamilnadu, India; g Department of Chemistry and Biochemistry, The Ohio State University, 151 W. Woodruff Ave, Columbus, OH 43210, USA; h Faculty of Science, Universiti Tunku Abdul Rahman, Jalan universiti, Bandar Barat, Kampar 31900, Malaysia; i Faculty of Health and Life Sciences, INTI International University, Nilai, 71800 Malaysia; j Faculty of Bioeconomics and Health sciences, University Geomatika Malaysia, Kuala Lumpur 54200, Malaysia; k Biomedical Research Unit and Lab Animal Research Centre, Saveetha Dental College, Saveetha Institute of Medical and Technical Sciences, Saveetha University, Chennai 602 105, India

**Keywords:** Aliphatic polymers, Biodegradable copolyesters, Drug delivery. biomedical applications, Sustainable Manufacturing

## Abstract

The study examined various properties of synthesized copolyesters PESC and PPSC. Inherent viscosities of the copolyesters, measured in 1,4-dioxane at 32 °C, were 0.65 dL/g for PESC and 0.73 dL/g for PPSC. Fourier-Transform Infrared Spectroscopy (FT-IR) revealed distinct absorption bands associated with ester carbonyl stretching, C–H bending vibration, C–H group symmetry stretching, and C–O stretching vibrations. ^1^H and ^13^C Nuclear magnetic Resonance (NMR) spectroscopy were used to identify specific protons and carbon groups in the polymer chain, revealing the molecular structure of the copolyesters. Differential Scanning Calorimetry (DSC) identified the glass transition, melting, and decomposition temperatures for both copolyesters, indicating variations in the crystalline nature of the copolymers. XRD Spectral studies further elaborated on the crystalline nature, indicating that PPSC is less amorphous than PESC. Biodegradation analysis showed that PESC degrades more quickly than PPSC, with degradation decreasing as the number of methylene groups increase. Scanning Electron Microscopy (SEM) images depicted the surface morphology of the copolyesters before and after degradation, revealing a more roughened surface with pits post-degradation. These findings provide comprehensive insights into the structural and degradable properties of PESC and PPSC copolyesters.

## Graphical abstract

**Figure:**
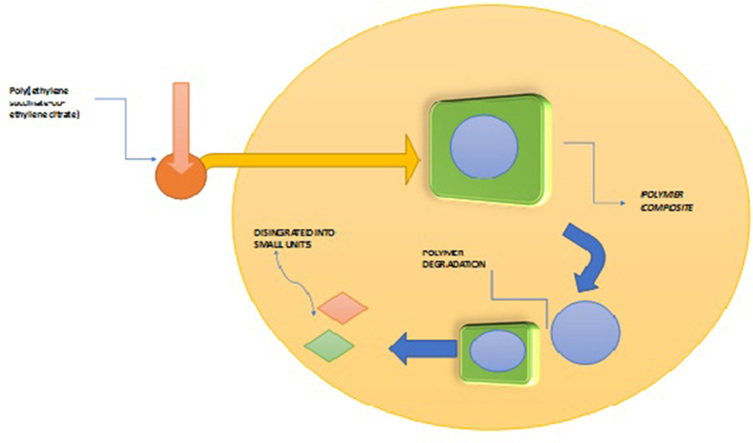

## 1 Introduction

Now a day's polymers turn into unavoidable materials in our day today life due to their applications enriched by their quality and comfort. This is attributed because of the versatile qualities of these polymers in terms of their strength, lightness, durability, protection and low value [[1], [2], [3]]. These days we find enormous application of polymers in industrial, technological and biomedical fields [4,5]. These polymers are considered as potential materials in innovative fields such as automobile, agriculture, medical, pharmaceutical, biomedical, electric and electronic science, information technology, etc. [[6], [7], [8], [9]]. In this scenario, a new version of polymer *viz.* biodegradable polymers were invented in order to control damage caused to the environment during their disposal of polymers. These new polymers easily decomposed by microorganisms. Synthetic polymers were initially developed for long-life and restrict to all or any types of degradation, together with biodegradation. A number of these merits of the compound have presently become attractive for suitable applications [[10], [11], [12], [13], [14]]. However from the future environmental preserving point of view it would be extremely supportive if these films were durable, rotten nearly on the attack of organisms over a period of time or they will force themselves to fragment as a result they are not going to become harmful to our atmosphere. Most of the polymers degrade in the way or alternative and hence these polymers are thought to be degradable [[15], [16], [17], [18]].

Aliphatic random copolyesters displays beneficial functions of biocompatibility and biodegradability, which are vital guidelines on artificial biodegradable aliphatic copolyesters. Hence it may be preferred in the selection of substances for *in vivo* applications. These days application of degradable polymers in medicine has received eminence with novel improvements in drug delivery systems. Mostly drug is appropriated all through the frame and it is not always limited to exact affected site. One potential answer for above concern is the usage of controlled drug delivery system, where the drug is discharged at a constant rate and feasibly cantered to the individual site [[19], [20], [21], [22], [23]]. In this scenario, current research work focuses on the synthesis and characterization of aliphatic random copolyesters with help of various diols. We propose to synthesis titanium tetra isopropoxide catalysed random copolyesters *viz.* Poly(ethylene succinate-*co*-ethylene citrate) PESC and Poly (propylene sebacate-*co*-propylene citrate) PPSC by direct melt polycondensation methodology. The polymeric structures, thermal behaviour and crystalline nature of synthesized copolyesters to be analyzed by various spectral techniques, X-ray diffraction pattern and scanning electron microscope [[24], [25], [26], [27], [28], [29]]. The biodegradation investigation of two random copolyesters to be evaluated from the results of biodegradation studies.

## 2 Materials and methodology

Ethylene glycol (E), Succinic acid (S), Citric acid(C), Sebacic acid (Se), and Propan-1,3-diol (P) were utilized as such after being bought from Sigma Aldrich. The catalyst, titanium tetraisopropoxide (TiTPo), was bought from Lancaster. The same grade of all other chemicals and solvents was utilized.

### 2.1 Synthesis of copolyesters

**Scheme 1:**
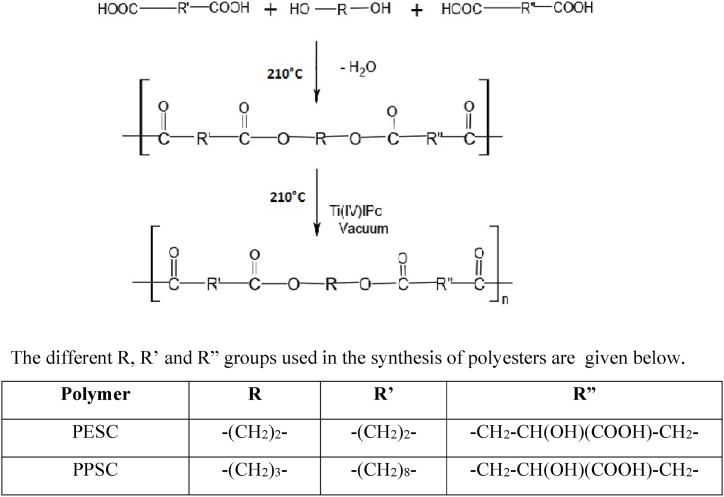
Synthesis of biodegradable copolyesters PESC and PPSC.

PESC and PPSC were combined to form the aliphatic copolyesters by a two-step melt polycondensation process. -A combination of 0.1 mol Succinic acid. To eliminate water as a byproduct, reaction flasks containing 0.1 mol of citric acid, 0.2 mol of Ethylene glycol, and 0.1 mol of TtiPO were gently heated to 160–210 °C for 2h. In order to raise the molecular weight of the polyester, the prepolymer was heated for 1h under vacuum. Methanol was used to separate the synthetic polymer. The polymer PPSC was created using the same process. Both polymers were vacuum-dried before being characterized. The following is the polyester synthesis plan (Scheme 1).

### 2.2 Characterization methods

The synthesized copolyesters had been characterized by ^1^H and ^13^C Nuclear magnetic resonance spectroscopy, Infrared spectroscopy, SEM and wide angle XRD pattern. The consistencies of copolyesters are determined in Ubbelohde viscometer with 1,4-dioxane as solvent. The Infrared spectra of the synthesized copolyesters recorded by Brucker optics Fourier Transform Infrared spectrometer using KBr pellets. ^1^H and ^13^C NMR spectra of synthesized copolyesters were observed in DMSO. Differential Scanning Calorimetry (DSC) identified the glass transition, melting, and decomposition temperatures for both copolyesters, indicating variations in the crystalline nature of the copolymers. The solubility of synthesized compounds were analyzed in different solvents. The XRD pattern determine the degree of crystallinity of the polymer. The morphology pattern of the copolyesters were analyzed by scanning electron microscopy. Further the biodegradable nature of synthesized copolyesters recorded and confirmed their efficiency.

## 3 Results and discussions

### 3.1 Viscosity of copolyesters

The inherent viscosities [30] of these random co polyesters were measured in 1, 4-dioxane at 32 °C and at a concentration of 20 mg/ml. The inherent viscosities values of PESC and PPSC are found to be 0.65 dL/g and 0.73 dL/g respectively.

### 3.2 Fourier – Transform Infrared spectroscopy

**Fig. 1:**
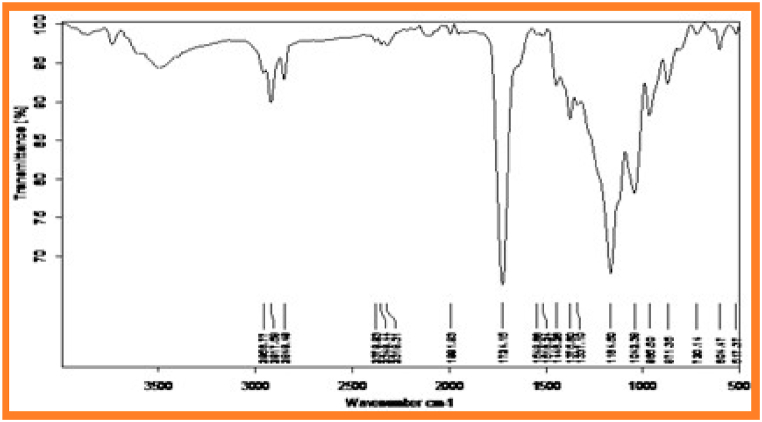
Infrared spectrum of PESC

**Fig. 2:**
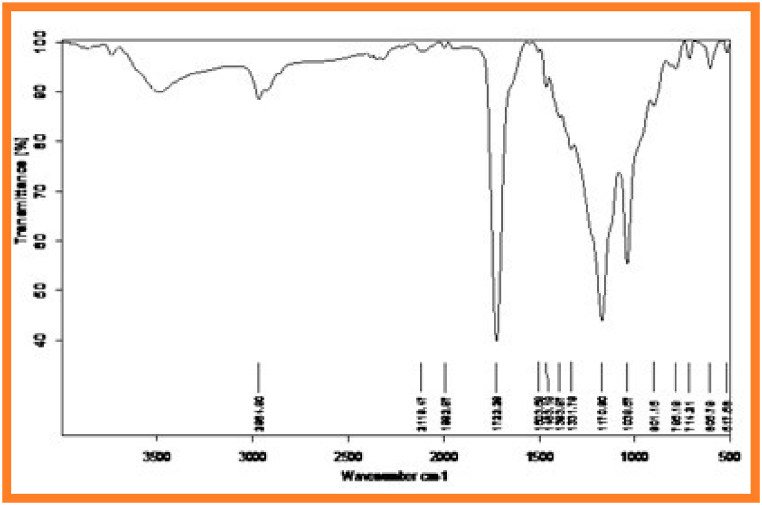
Infrared spectrum of PPSC.

Fig. 1 and 2 represents FT-IR spectrum of synthesized copolyesters PESC and PPSC. The absorption band appeared PESC at 1724 cm^−1^ and PPSC at 1721.29 cm^−1^ are related to ester carbonyl stretching frequency [31]. The peaks at 1042.38 cm^−1^ and 1038.67 cm^−1^ are corresponding to C–H bending vibration. Symmetry stretching frequency of aliphatic C–H group appeared at 2966.77 cm^−1^ and 2984.08 cm^−1^. Stretching vibrations of C–O denoted peaks at 1164.50 cm^−1^ and 1170.90 cm^−1^.

### 3.3 ^1^H Nuclear Magnetic Resonance spectral data of copolyesters PESC and PPSC

**Fig. 3:**
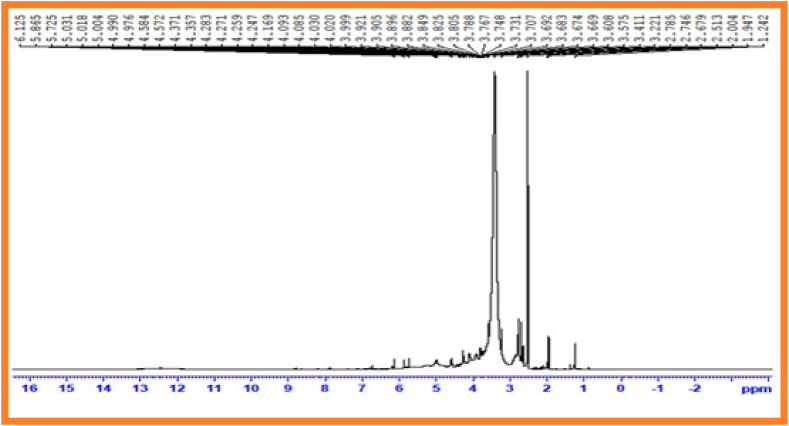
^1^H Nuclear magnetic resonance spectrum of PESC

**Fig. 4:**
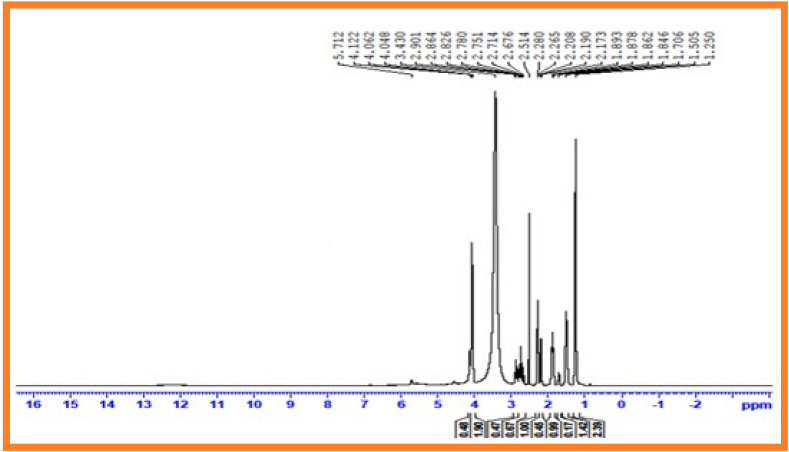
^1^H Nuclear magnetic resonance spectrum of PPSC.

In ^1^H nuclear magnetic resonance spectroscopy was used to analyse the structure of repeating gadgets and type of proton that exist in polymer chain [32]. ^1^H NMR spectrum of synthesized copolyesters PESC and PPSC represented in Fig. 3, Fig. 4. The peak at 3.20 ppm and 3.25 ppm corresponds to methylene protons of acids. Central methylene proton of diols were denoted peaks at 2.1 ppm and 2.0 ppm. Terminal methylene proton of diols were represented peaks at 1.1 ppm and 1.2 ppm.

### 3.4 ^13^C Nuclear Magnetic Resonance spectral data of copolyester of PESC and PPSC

**Fig. 5:**
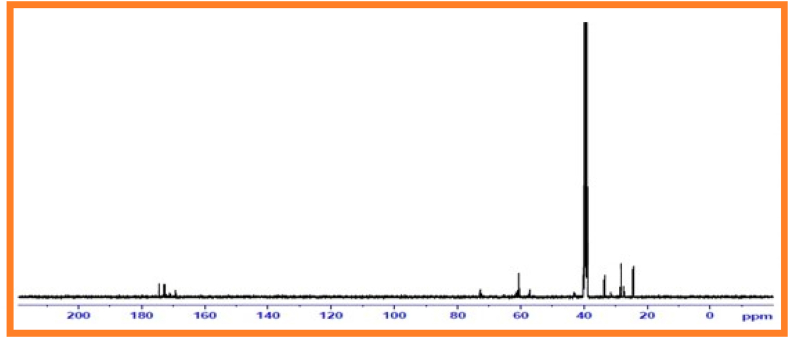
^13^C Nuclear magnetic resonance spectrum of PESC

^13^C NMR Spectroscopy examinesrepeating groups present in aliphatic random copolyesters [33].^13^C Nuclear magnetic resonancespectrum of PESC presented in Fig. 5. Carbonyl carbon appears as a peak at170.0 ppm. An intense peak at 61.0 ppm is attributed to -*O*–CH_2_– methylene carbon connected with oxygen. The dicarboxylic acid unit display peaks at 24.0 ppm.

**Fig. 6:**
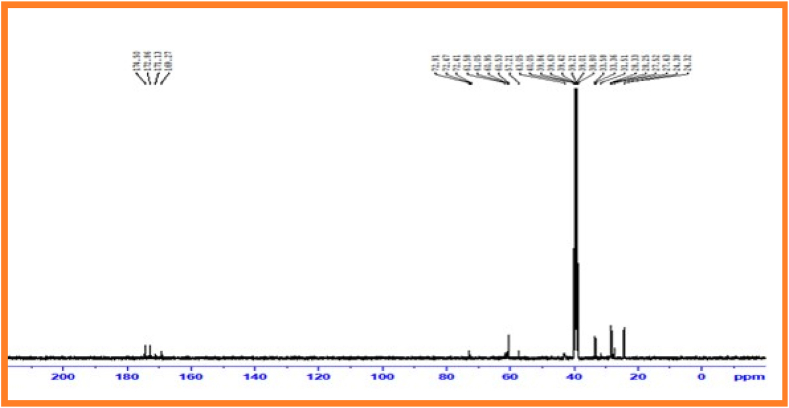
^13^C nuclear magnetic resonance spectrum of PPSC.

The ^13^C NMR spectroscopy of PPSC examines repeating groups present in aliphatic random copolyesters. ^13^C Nuclear magnetic resonance spectrum of PPSC given in Fig. 6. Carbonyl carbon appears as a peak at 172.86 ppm. An intense peak at 60.53 ppm is attributed to -*O*–CH_2_– methylene carbon connected to oxygen. The dicarboxylic acid unit display peaks at 31.51 ppm.

### 3.5 Differential Scanning Calorimetry (DSC)

**Fig. 7:**
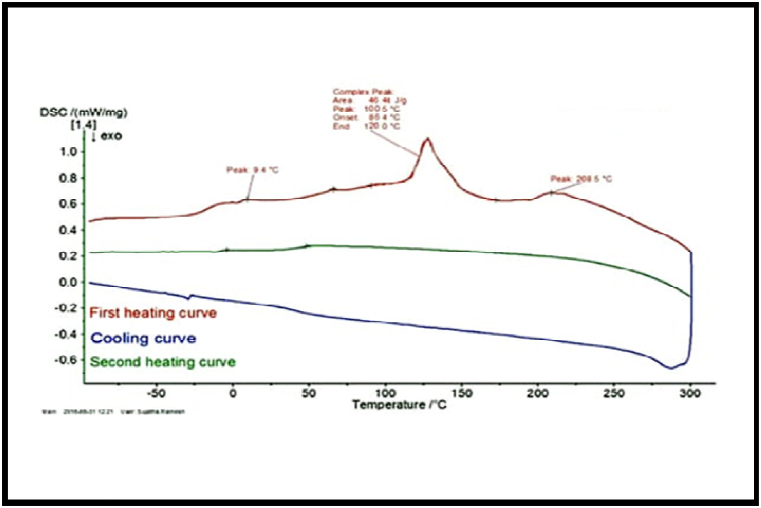
DSC Thermograms of polyesters Spectrum of PESC.

**Fig. 8:**
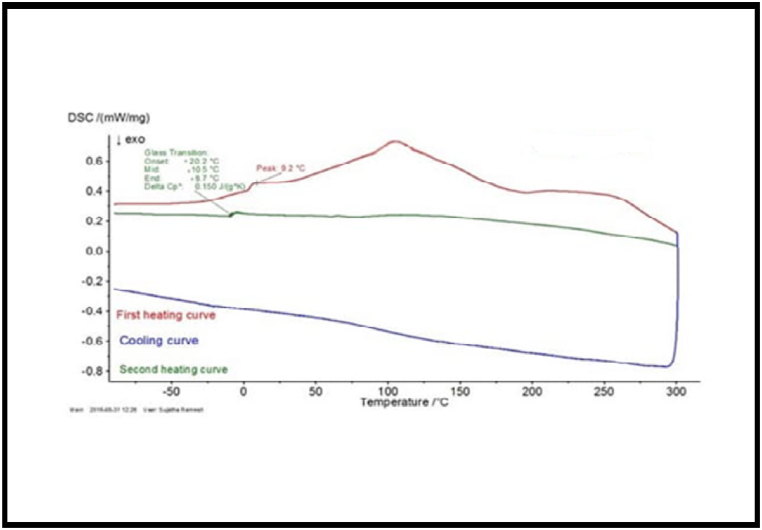
DSC Thermograms of polyesters Spectrum of PPSC.

The DSC thermograms of the two biodegradable copolyesters PPSC and PESC are presented in Fig. 7, Fig. 8. These thermograms show glass transition temperature (Tg) at −16.5 °C, and −15.5 °C melting temperature (Tm) at 48.12 °C and 45.2 °C decomposition temperature (Td) at 310.02 °C and 304.02 °C for the polyesters PPSC and PESC respectively (Table 1). It is worth noting that the melting temperature, Tm increases with increase in the number of methylene group in the repeating unit of the polymer chain. It is observed from DSC data of the copolyesters that polyester PESC exhibits the lower melting and glass transition temperature while these polyester less crystalline nature.

**Table 1:** Thermal Data of copolyesters PESC and PPSC.

S.No	Polymers	Tg (0C)	Tm (0C)	Td (0C)
1.	PESC	−15.5 °C	45.2 °C	304.02 °C
2.	PPSC	−16.5 °C	48.12 °C	310.02 °C

### 3.6 XRD Spectral Studies of PESC and PPSC

**Fig. 9:**
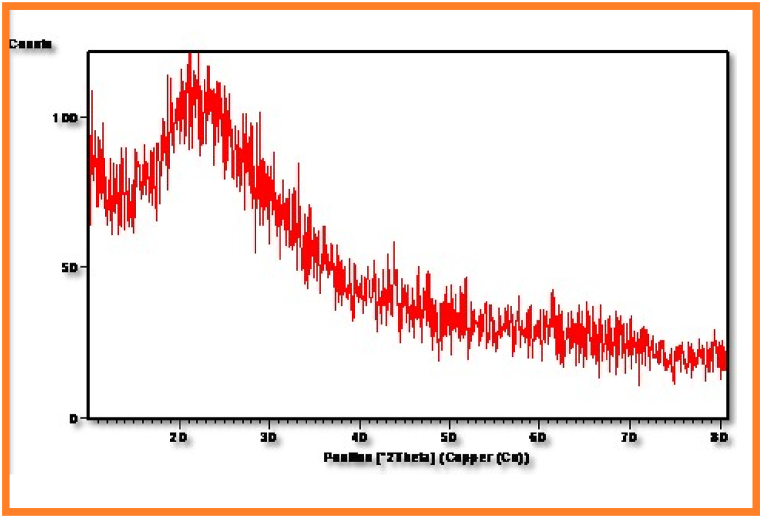
Xrd patterns of copolyesters PEMC

**Fig. 10:**
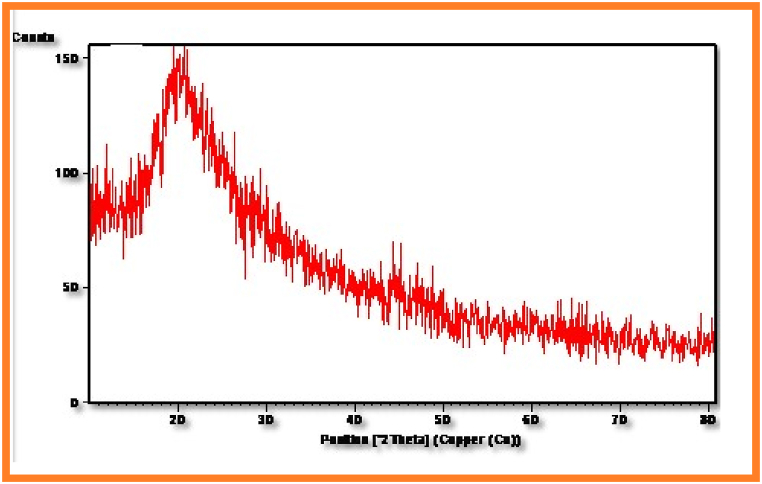
Xrd patterns of copolyesters PPSC.

The X-ray diffraction grams of PESC and PPSC were exhibited in Fig. 9, Fig. 10. By employing Scherrer equation, size of the particles were determined using X-ray diffraction studies. Polycrystalline materials can be examined by XRD analysis [34]. In general, during analysis, the sample is exposed to a collimated X-ray beam, with position of the type and power of dispersing by arranging in parallel nuclear planes of the sample, at explicit points.

By employing this technique, the nature of the compound, crystallinity degree and direction, crystalline stage, and size of the crystallites can be distinguished(<b>Table-2)</b>. Sharp and wide diffraction are observed for crystalline and shapeless materials, individually. This reveals that the crystalline nature of synthesized copolyesters rises with the number of bendy segments. From the XRD pattern, it's found that PPSC is less amorphous than PESC.

**Table-2:** XRD spectral studies of PESC and PPSC.

Sl.No	Polymers	Pos. [^o^2Th. ]	Height [ cts ]	FWHM Left [^o^2Th. ]	d-spacing [ Å ]	Rel. Int. [ % ]
1.	PESC	19.4586	30.4432	1.2002	3.51622	100.00
2.	PPSC	34.3266	32.5868	1.3296	3.58428	100.00

### 3.7 Biodegradation analysis of copolyesters

The synthesized copolyester PEMC and PPSC thin films of area 10 × 10 mm^2^ and 200 μm thickness had been employed in a petri-dish consisting 10 mL of phosphate buffer solution. After specific time intervals of incubation, the films had been expelled from dish, several times washed with distilled water, dried and weighed till steady weight is acheived [35]. The procedure was continued at particular time intervals.

**Fig. 11:**
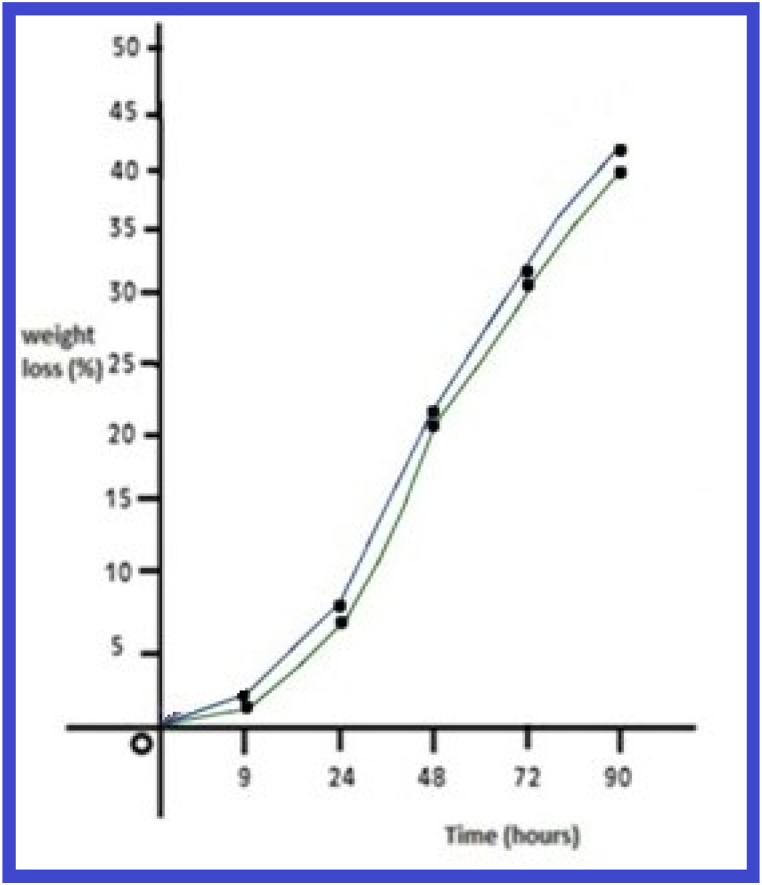
Biodegrading ability graph of copolyesters PEMC and PPSC.

The weight loss percentage of co polyesters PEMC and PPSC plotted against function of time (Fig. 11). PESC exhibit higher weight loss than PPSC. The weight loss percentage to polyester PESC and PPSC during hydrolysis by phosphate buffer is represented in Table 3.

**Table 3:** Weight loss percentage of polymers PESC and PPSC.

S.No	Time (hours)	Weight loss percentage	
		PESC	PPSC
1.	00	0.0	0.0
2.	09	3.6	3.2
3.	24	9.6	9.0
4.	48	24.8	22.2
5.	72	34.8	32.2
6.	90	46.8	42.6

From the biodegradation test results, it is understood that the rate of biodegradation decreases with increasing number of methylene groups as a result the copolyesters PEMC, are found to be highly degraded compared with the other prepared copolyesters such as PPSC.

#### 3.7.1 SEM analysis of PEMC and PPSC

**Fig. 12:**
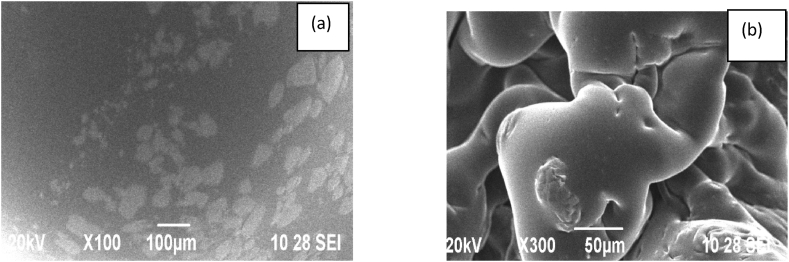
(a) Scanning Electron Micrographs before degradation, (b) after degradation of copolyesters PESC.

**Fig. 13:**
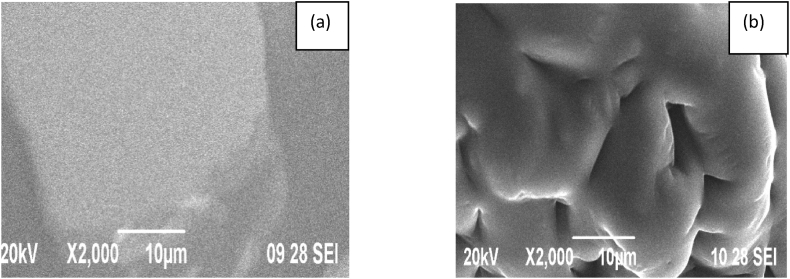
(a) Scanning Electron Micrographs before degradation, (b) after degradation of polyesters PPSC.

The structure morphology of copolyesters PEMC and PPSC were investigated by Scanning Electron Microscopy studies [35]. The electron micrograph of copolyesters biodegradation of PESC and PPSC in presented as Fig. 12, Fig. 13. The SEM images in Fig. 12, Fig. 13 a represent the mono-dispersed smooth surface of the co-polyesters and Fig. 12, Fig. 13b represent the biodegraded surface of the co-polyesters, respectively.

SEM images of the before and after in Fig. 12, Fig. 13 show an unpleasant surface with pits on the film, which is ample proof for the corruption property for the blended copolyesters. The surface morphology of the new unpredictable copolyester was broken down through sifting electron amplifying instrument.

## 4 Conclusion

After a thorough examination of the copolyesters PESC and PPSC, distinct features emerged. In terms of inherent viscosities, PESC registered at 0.65 dL/g while PPSC stood at 0.73 dL/g. Their chemical and structural properties were confirmed by both FT-IR and NMR spectroscopic methods. Thermally, DSC analyses indicated that PESC's transition temperatures were marginally lower than those of PPSC, influenced by the methylene group quantity. XRD investigations also pointed out that PPSC had a more crystalline nature, whereas PESC was more amorphous. Biodegradation studies suggested that PESC degrades more swiftly, a feature further supported by 10.13039/100014281SEM images displaying surface inconsistencies after biodegradation. Taken together, these insights underscore the distinct qualities of each polyester and their promising roles in biodegradable fields.

## 5 Institutional review Board Statement

Not applicable.

## Informed Consent Statement

Not applicable.

## CRediT authorship contribution statement

**A. Kesavan:** Conceptualization, Data curation, Formal analysis. **T. Rajakumar:** Conceptualization, Data curation, Formal analysis, Investigation. **M. Karunanidhi:** Formal analysis, Investigation, Methodology. **A. Ravi:** Conceptualization, Data curation, Investigation, Project administration. **P.A. Vivekanand:** Conceptualization, Data curation, Formal analysis, Supervision, Validation. **P. Kamaraj:** Validation, Visualization, Writing – original draft, Writing – review & editing. **Natarajan Arumugam:** Funding acquisition, Investigation, Methodology, Visualization, Writing – review & editing. **S. Hari Kumar:** Data curation, Formal analysis, Methodology, Resources. **Karthikeyan Perumal:** Formal analysis, Investigation, Methodology, Writing – review & editing. **Sinouvassane Djearamane:** Writing – review & editing, Validation, Visualization. **Mohammod Aminuzzaman:** Writing – review & editing, Investigation, Methodology. **Ling Shing Wong:** Formal analysis, Funding acquisition, Investigation. **Saminathan Kayarohanam:** Methodology, Resources, Software, Supervision.

## Declaration of competing interest

The authors declare that they have no known competing financial interests or personal relationships that could have appeared to influence the work reported in this paper.
